# Group virtual nutrition and teaching kitchen intervention versus time/attention-matched health education curriculum control to improve dietary quality and social engagement among older Veterans with impaired mobility: a randomized controlled trial

**DOI:** 10.1186/s12877-026-07556-x

**Published:** 2026-04-28

**Authors:** Bailey Capra, Odessa Addison, Jason R. Falvey, Lisa Juckett, Steven J. Prior, Brock A. Beamer, John Sorkin, Jamie Giffuni, Jeffrey Beans, Elizabeth A. Dennis

**Affiliations:** 1 Geriatric Research, Education, and Clinical Center (GRECC), Veterans Affairs Maryland Health Care System (VAMHCS), 10 North Greene Street, Baltimore, MD 21201 USA; 2 Department of Physical Therapy and Rehabilitation Science, University of Maryland School of Medicine, 100 Penn Street, Allied Health Research Building (AHRB), Baltimore, MD 21201 USA; 3 School of Health and Rehabilitation Sciences, College of Medicine, The Ohio State University, 453 West 10th Avenue, Columbus, OH 43210 USA; 4 Department of Kinesiology, University of Maryland School of Public Health, 4200 Valley Drive, College Park, MD 20742 USA

**Keywords:** Virtual nutrition education, Virtual teaching kitchen, Dietary quality, Mobility, Frailty, Social isolation

## Abstract

**Background:**

Older Veterans with impaired mobility often face barriers to food shopping, meal preparation, and cooking that can contribute to poor diet quality and declining health.

**Methods:**

This randomized controlled trial will test whether a 12-week virtual nutrition and teaching kitchen program improves diet quality (Healthy Eating Index [HEI]), compared with time- and attention-matched contact-control group. We plan to enroll 180 Veterans *≥* 65 years of age with mobility limitations who will be randomized to an intervention or control group. Participants in the intervention group will attend one live, one-hour virtual session each week for 12 weeks, including cooking demonstrations, and tailored nutrition education, with adaptive equipment and occupational therapy guidance provided as needed. Control participants will attend matched group health education sessions focused on healthy aging. Outcome assessments occur at baseline, 3 months, and 6 months. The primary outcome is change in HEI score from baseline to 3 months. Secondary outcomes include change in HEI from 3 to 6 months, and changes in social isolation, loneliness, and health-related quality of life, physical function, body composition, and fear of falling from baseline to 3 months. Sample size calculations indicate that 73 participants per group will provide 80% power to detect a 5.5-point between-group difference in HEI, allowing for 20% attrition.

**Discussion:**

This trial aims to provide evidence on effectiveness of a virtual nutrition and cooking program designed to improve diet quality and social connection in older adults with mobility impairments. We anticipate that this protocol will show that virtual nutrition education and cooking demonstrations is an efficacious strategy to improve diet that also has potential for scalability through implementation into existing health promotion programs.

**Trial registration:**

This trial was registered at ClinicalTrials.gov: NCT06726083 on 12/05/2024 (https://clinicaltrials.gov/study/NCT06726083).

## Highlights

Two-arm RCT (*N* = 180; ≥65 years with mobility limitations) testing a 12-week virtual nutrition + teaching kitchen program (with produce delivery) vs. time/attention-matched health education curriculum control.

Primary outcome is change in Healthy Eating Index (HEI) with assessments at baseline, after the 3-month intervention, and at 3 months post-intervention. Secondary outcomes include food literacy, health literacy, food insecurity, social isolation, health-related quality of life, frailty, body composition, and physical function.

6-month follow-up focus groups will assess sustained effects of the intervention.

## Background

There are > 8 million older Veterans (> 65 years) in the United States, with nearly half self-reporting having a disability [1] such as impaired mobility. Disability is a well-established risk factor for social isolation [2–4] and inadequate nutrition [5–9] Veterans also demonstrate an increased risk for obesity and multimorbidity than non-Veterans [10–12], and are more likely to experience social isolation [1] which can negatively impact dietary quality [13–15]. Conversely, poor dietary intake contributes to chronic disease risk and loss of muscle mass and strength [16], which limits function, decreases mobility, and exacerbates fall risk [17–19] Maintaining safe mobility with aging is critical to mitigate risk, healthcare utilization, and expenditures [20].

Healthy diets with fruits and vegetables lower the risk of chronic diseases, including mobility disability [21], and are associated with higher muscle mass, strength, and physical performance, which reduces the rate of mobility decline [22] Cross-sectional studies demonstrate that dietary patterns characterized by a higher intake of fruits, vegetables, and unsaturated oils are associated with lower rates of disability [23, 24], frailty [25], and greater balance than those with less optimal intake [22] Despite this, over 50% of older adults do not consume diets that align with national recommendations [26], including older Veterans with impaired mobility [27] Further, few studies have explored how intentionally improving dietary quality may be associated with positive changes in physical functioning through randomized controlled trials.

Among older adults with mobility disability, common self-care tasks like food shopping, meal preparation, and cooking are barriers to consuming a healthy diet, resulting in poor dietary intake. Mitigating some of these factors could make meaningful differences in Veterans’ diet quality [28] Group-based interventions are a potential strategy to provide social connectedness and mutual support, both powerful factors for older adults as frequent contact may be beneficial to reduce the risk for functional impairments [29] and improve health-related quality of life [30]. Previous research demonstrated that in-person participation in once-monthly group classes for older Veterans resulted in increases in self-reported dietary quality [31], but mobility limitations may reduce participation in social group activities [32] Thus, transitioning to a virtual platform may increase accessibility of group nutrition classes to those with limited transportation options and/or those with mobility limitations while still reducing social isolation [33] In this randomized controlled trial, we will determine if implementing a 3-month virtual group nutrition intervention paired with virtual cooking demonstrations and weekly produce delivery tailored for older Veterans with impaired mobility will improve diet quality and thus, functional mobility versus a contact control group. We hypothesize that virtual group nutrition education classes and cooking demonstrations, personalized to include considerations of physical limitations, age-related changes in taste, and technological barriers, will result in favorable improvements in dietary quality and functional mobility while maintaining social interaction.

## Methods

### Study design and setting

**Fig. 1:**
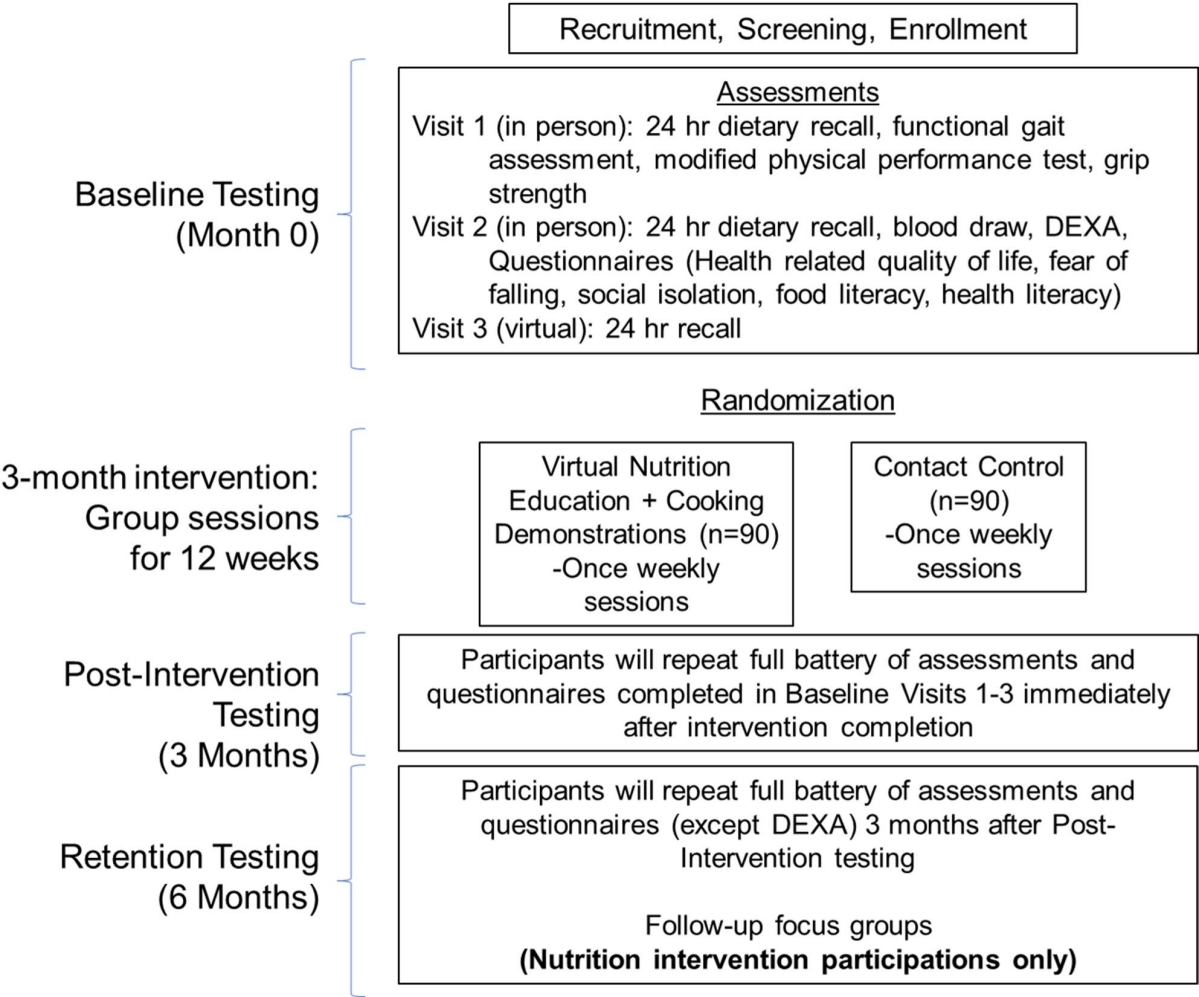
Experimental design

We anticipate that this two arm, parallel randomized controlled trial will be completed over ~ 8 months per participant. An overview of the study is summarized in Fig. 1.

The first visit will consist of a 24-hr dietary recall, functional gait assessment, and modified physical performance test (MPPT). The second visit will be at least two days later and will consist of a second 24-hr recall, health-related quality of life, fear of falling, social isolation, food literacy, and health literacy questionnaires, collection of fasting blood sample and dual-energy x-ray absorptiometry (DEXA) scan to assess body composition. A third diet recall will be completed virtually [34]. All study procedures except for the DEXA will be repeated in an identical pattern at 3 months and 6 months (3 months after intervention completion). DEXA scans will occur at baseline and 3 months. Participants will receive $25 upon completion of each assessment timepoint (baseline, 3-months, and 6-months). Participants in the nutrition intervention group may receive an additional $20 for participation in the follow-up focus group ($95 total). This study protocol is approved by the institutional review board of the University School of Medicine and the VA Research and Development Committee (HP 00112264, version 3, Amendment 2 (2/19/2026)). The study is registered at *ClinicalTrials.gov* (NCT06726083).

### Participants and eligibility

Veterans > 65 years with reduced gait speed (< 1.0 m/s), elevated four square step test time, (> 12 s), or use of an assistive device, who also indicate that they would like to improve their eating habits (defined by response of ≤ 3 on a scale of 1 to 5 to the question, ‘Do you consider your eating habits to be healthy’) will be recruited from the Baltimore greater metro area. The study coordinator will complete a telephone screen to assess additional eligibility of interested participants (Table 1). Those who pass the phone screening will be invited to sign informed consent. The consent form includes information explaining how collection and use of participant data and biological specimens may be used in future research studies and will not be sold or used for the production of commercial products. After providing written informed consent and HIPAA (Health Insurance Portability and Accountability Act) authorization, participants will complete baseline assessments. Participants may continue usual medical care and stable medications during the study. Any new treatments or changes to existing care will be documented and evaluated for potential impact on study outcomes.

**Table 1:** Eligibility criteria

Inclusion Criteria	Exclusion Criteria
- Veterans > 65 years - Reduced gait speed (< 1.0 m/s) - Elevated four square step test (> 12 s) or use of an assistive device - Want to improve their eating habits - Access to an internet accessible device	- Uncontrolled diabetes mellitus (HbA1c > 10) or current renal replacement therapy (e.g. dialysis) - Other medical condition precluding patient participation in this study as per medical judgement of the study team - Dementia as self-reported or found on medical record review - Currently participating in a diet or weight loss intervention - Volatile behavioral issues or unable to work successfully in a group environment/setting

### Recruitment

Participants will be recruited using the Department of Veterans Affairs Informatics and Computing Infrastructure (VINCI), the University of Maryland Baltimore Pepper Center Registry, and from the Baltimore metropolitan area by media advertisement. Those referred to the Gerofit Program will also be approached about their interest in participating in the study. Acceptable recruitment is defined as 80% of total targeted enrollment for the lifespan of the study. Retention will be assessed by frequencies of drop-outs; 80% retention will be considered acceptable.

### Intervention

Following baseline testing, participants will be randomized to one of two virtual intervention groups using a 1:1 ratio (nutrition intervention; *n* = 90 or contact control; *n* = 90) using blocked randomization with a 1:1 allocation ratio. Randomization is not stratified. The randomization allocation, generated by the study statistician, will be implemented using the Randomization module within the study database in VA REDCap. The Randomization module within REDCap prevents all users from viewing the treatment assignment of future study participants. This will ensure that all study personnel are blinded to treatment assignment prior to randomizing each participant.

The study coordinator will work with participants individually to ensure technology access and increased comfort level using the virtual platform. Devices and/or internet access will be provided to participants who report this as a barrier to participation. For both the nutrition intervention and contact control groups, we will enroll up to 15 participants into each virtual class cohort and run multiple different group sessions.

We will define successful adherence as completion of > 80% of all class sessions by the majority of participants. Length of time required for each educational session as well as modifications to the sessions will be tracked according to the Core functions and forms framework [35]. The core functions of the nutrition education protocol include 1) each session is led by a trained facilitator, 2) each session follows a standardized lesson plan, 3) each session concludes with question and answer opportunities with participants, and 4) each session is held in a group-based format. The “forms” are the specific ways in which each of the core functions are carried out when implemented under real world conditions and are modified to meet the needs of the local context and participants. For instance, standardized lesson plans may be adapted based on the cultural background of participants, and question and answer opportunities may include questions that are unique to a certain patient population (e.g., people with heart disease). All “forms” will be tracked by the class facilitator during each session to explore how the intervention should be tailored to meet participants needs and inform how other organizations may want to implement our nutrition program in the future.

Participants may discontinue at any time based on clinical judgement in response to adverse events, changes in health status, or at the participants request. Harms / adverse events are defined as any unfavorable, unintended sign, symptom, or disease associated with study participation, and will be systematically monitored throughout the study and recorded in study documentation.

#### Virtual nutrition education + produce group

In the nutrition intervention group, 1-hour live virtual group sessions led by a trained study team member will be held weekly over 12 weeks using a HIPAA-compliant online platform.

The first part of class of each week will include the teaching kitchen cooking demonstration (~ 30 min). The remainder of the class will focus on dietary education and discussion. An overview of the nutrition and cooking skills education topics are presented in Table 2. Dietary education will review basic cooking skills, (e.g., food safety; preparation techniques such as washing fruits and vegetables) and progress to content on food selection, preparation, consuming adequate protein sources to maintain muscle mass, flavor enhancement with spices/herbs, and mindful eating. If needed, participants may also receive cooking equipment such as adaptive cooking utensils, small appliances like crock pots and/or toaster ovens/air fryers to allow participants to prepare meals in an area with a limited amount of space. Each week participants will receive an allotment of produce, similar to a community supported agriculture model [36]. In this format, participants will receive a pre-selected number of locally-grown produce items each week during the intervention provided by a local farm.

**Table 2:** Overview of Nutrition and Cooking Skills Education Topics

- Review of food safety: good hygiene, cooking temperatures, knife safety; How to make your kitchen environment work for you.
- Proper food storage; Washing fresh produce; Reducing food waste.
- Balance/body alignment tips during food prep.
- Overview of healthy plates and food groups.
- Importance of protein for aging Veterans.
- Balancing proteins, carbohydrates & fats.
- Grocery shopping; Eating well on a budget.
- Healthy, quick breakfasts, lunches, dinners and snacks.
- Eating with mindfulness; Addressing hunger & fullness.
- Healthy recipe substitutes and modifications.
- Cooking with spices/herbs to enhance flavor.
- Home safety in the kitchen and dining spaces.

Classes will also incorporate virtual home safety assessments from an occupational therapist who will give recommendations to adapt the home environment to reduce risk of falling and improve physical functioning. Assessments will be completed using an adapted version of the American Occupational Therapy Association’s ‘Safe at Home’ Checklist [37]. Recommendations include proper body mechanics while preparing food, strategies to overcome fatigue that might result from or discourage meal prepping, tips to improve strength for lifting heavier pots and pans, proper lighting in the kitchen and dining areas, safe modifications when cutting produce or preparing meals, etc. For home safety concerns that are discovered during participants’ involvement in the intervention, we will refer participants to VA rehabilitation services and work with participants’ local Area Agency on Aging whose staff can coordinate home repair services or more in-depth home safety assessments.

The class will also provide an opportunity for group discussion, with the goal of providing participants with a variety of meal planning and recipe options and to expose them to new recipes and foods to broaden their options for healthy choices. This will also serve to introduce participants to each other and allow them to expand their social support network, discuss barriers to healthy eating, and exchange cooking techniques and recipes.

#### Contact control group

In the contact control group, virtual live group health education sessions led by staff will be held for one hour/week over 12 weeks. The content for the program was modeled on the “10 Keys to Healthy Aging” curriculum and the National Council on Aging’s “Aging Mastery Program”. Topics focus on a broad range of healthy aging, such as importance of sleep, physical activity, goal setting, self-care, and common comorbidities [38, 39].

### Outcomes

Table 3 outlines the study outcomes and timepoints of assessment.

**Table 3:** Outcomes and Timepoints Measured

Assessment/Outcome	Baseline			Post-Intervention			Retention-Testing		
	V1	V2	V3	V1	V2	V3	V1	V2	V3
24-hr Dietary Recall/HEI	*X*	*X*	*X*	*X*	*X*	*X*	*X*	*X*	*X*
Grip Strength	*X*			*X*			*X*		
Height and Weight	*X*			*X*			*X*		
Modified Physical Performance Test	*X*			*X*			*X*		
Functional Gait Assessment	*X*			*X*			*X*		
Cooking and Food Skills Measure		*X*			*X*			*X*	
BRIEF Health Literacy Screening Tool		*X*			*X*			*X*	
USDA Adult Food Security Survey Model, 6-Item Short Form		*X*			*X*			*X*	
Lubben Social Network Scale		*X*			*X*			*X*	
Life Space Assessment		*X*			*X*			*X*	
UCLA Loneliness Scale		*X*			*X*			*X*	
PROMIS Global Health		*X*			*X*			*X*	
CHAMPS Activities Questionnaire for Older Adults		*X*			*X*			*X*	
DEXA		*X*			*X*				
Blood Draw		*X*			*X*			*X*	
Falls-Efficacy Scale-International		*X*			*X*			*X*	
Follow-up Focus Group							*X*		

Timepoints of study assessments. Each timepoint consists of 3 visits (V1, V2, V3) over a two-week period

#### Dietary quality

The primary outcome of the study is dietary quality. To capture habitual dietary intake, participants will complete three 24-hr recalls [34] via the online Automated Self-Administered 24-hr (ASA24) Dietary Assessment Tool, developed by the National Cancer Institute, Bethesda, MD. Recalls will be collected for two week days and one weekend day within a two-week period. The dietary recalls will be used to calculate diet quality using the HEI; scores range from 0 to 100; higher scores indicate better adherence to the Dietary Guidelines for Americans.

#### Questionnaires

Secondary outcomes will include measures of food literacy, health literacy, food insecurity, social engagement and health-related quality of life. Food literacy will be evaluated using the Cooking and Food Skills measure which assesses confidence and knowledge of cooking and home meal preparation [40], as well as the NCI’s Food Attitudes and Behavior Survey, which determine factors associated with fruit and vegetable intake, food purchasing patterns, distance to local food stores, frequency of grocery delivery and sources of meals [41]. These scales have good internal consistency reliability with Cronbach’s alphas ≥ 0.68. Health literacy will be assessed with the BRIEF Health Literacy Screening tool [42], a 4-item instrument that evaluates: (1) How often do you have someone help you read hospital materials? (2) How confident are you filling out medical forms by yourself? (3) How often do you have problems learning about your medical condition because of difficulty understanding written information? and (4) How often do you have a problem understanding what is told to you about your medical condition? The summative score ranges from 4 to 20; inadequate (4–12); marginal (13–16); and adequate (17–20). These questions were found to be effective in detecting inadequate health literacy. Food insecurity will be measured using the USDA Adult Food Security Survey Model, 6-Item Short Form, to assess the level of food security in the previous year (> 2 affirmatives indicate food insecurity; >5 indicate hunger) [43]. This tool has a sensitivity of 92.0% and specificity of 99.4%.

The study also aims to evaluate outcomes related to social connectedness. Social isolation will be measured using the Lubben Social Network Scale [44], a 12-item scale to assess self-reported social engagement including family and friends (Cronbach’s α = 0.70), and the UCLA Loneliness Scale [45], a 20-item scale to measure subjective feelings of loneliness & social isolation (Cronbach’s α 0.89 < 0.94). Health-related quality of life (HRQoL) will be assessed with the PROMIS Global Health (v1.2) [46] which consists of 10 items on a 5-pt Likert scale to measure an individual’s physical (GPH) and mental health (GMH). The GPH score comprises 4 items on physical health, physical functioning, pain intensity, and fatigue. The GMH score includes 4 items on overall HRQoL, mental health, satisfaction with social activities/relationships, & emotional problems.

#### Anthropometrics

Body height will be measured by a stadiometer to the nearest ¼ inch without shoes, and body weight will be measured to the nearest ½ pound in light clothing without shoes using a beam balance scale. Height and weight will be used to calculate BMI (kg/m^2^). Waist circumference will be measured using a standard tape measure. Total and regional percentage body fat, absolute fat mass and fat-free mass will be measured using dual energy X-ray absorptiometry (DEXA).

#### Functional outcomes

We will evaluate changes in frailty and physical function. The physical frailty phenotype consists of five criteria: weakness as measured by grip strength, self-reported exhaustion, unintentional weight loss (10 or more pounds in the past year), slow gait speed, and low physical activity [47]. Frailty is defined as meeting three or more of the five criteria [47]. This physical frailty phenotype has been shown to be a predictor of falls, worsening mobility, morbidity, and mortality [47]. Grip strength will be measured with a dynamometer, and physical activity will be calculated using the “CHAMPS Activities Questionnaire for Older Adults” [48]. Physical function will be evaluated using the Modified Physical Performance Test (MPPT), which is a nine-item standardized test used to identify frailty and mobility dysfunction in older individuals [49]. It scores individuals on the time it takes to complete such tasks as donning and doffing a coat, picking up a penny from the ground, and ascending the stairs. These scores are then summed out of 36 and scores of less than 32 indicate at least mild frailty [49] The MPPT has high inter-rater reliability (ICC = 0.94–0.99) [50]. The Functional Gait Assessment (FGA) will be used as a measure of mobility. The FGA is a 10-item clinical gait test that is based on the dynamic gait index (DGI). It tests higher level dynamic gait activities that are part of daily functional mobility such as turning safely and ambulating backwards. Total scores ranging from 0 to 30 and scores below 22 are predictive of future falls. The FGA has excellent inter-rater reliability (ICC=0.93) and predictive validity for falls [51] The Falls-Efficacy Scale-International (FES-I) will be administered to determine participants’ concern relative to their risk of falling – a frequent occurrence among older adults with mobility impairments. The FES-I is a 16-item self-reported tool that measures fear of falling when completing routine activities such as preparing a meal, going shopping, getting dressed, visiting with friends and family, and reaching up or bending down [52]. It has excellent internal consistency as well as test-retest reliability (Cronbach’s α = 0.96; ICC = 0.96) [53] and is scored from 16 to 64 points (high fear of falling > 23 points) [54].

#### Blood draw

Blood draws will be collected by a trained health worker in a quiet, clean, well-lit area. Participants will be asked to fast overnight (~ 8–12 h) prior to blood sampling. We will store plasma and serum and blood cells for analysis. We plan to measure biomarkers of dietary changes and cardio-metabolic risk factors. We may measure new hormones, inflammatory proteins and biomarkers as other technologies for measuring biomarkers of aging-related diet and muscle health are discovered.

#### Follow up focus groups

Participants randomized to the nutrition intervention who completed all outcome assessments will be invited to participate in focus groups at 6 months. We will complete 3–4 focus groups with ~ 5–8 participants per focus group (total *N* = 20). Sessions will be recorded, transcribed, and coded using qualitative software to identify themes, examine patterns and overlap, and develop a network diagram of interrelationships. Themes will describe correlates of diet quality, social engagement, and technology use that likely influence treatment. To inform future implementation and sustainment, transcripts will also be coded for implementation barriers and facilitators, and mapped to the Consolidated Framework for Implementation Research (CFIR) [55, 56]. These follow-up focus groups will provide key information on sustainability and discern barriers and facilitators to virtual intervention implementation and continued adherence after intervention completion. Key questions for the follow-up focus group include: (1) How did this program influence your ability to move safely around your home?; (2) How did this program influence your social opportunities?; (3) How did this program help you prepare meals at home?

### Sample size and power

Group sample sizes of 73 in the experimental arm and 73 in the control arm achieve 80.3% power to reject the null hypothesis of equal mean changes in the HEI2015 total score when the population difference in mean change in the two arms is µ1 - µ2 = 5.5 with a standard deviation for both arms of 13.3 and with a significance level (alpha) of 0.05 using a one-sided two-sample equal-variance z-test. The standard deviation is derived based on work from our nonrandomized virtual nutrition education with cooking demonstrations pilot study data. We anticipate enrolling 90 participants per group to achieve this target sample size assuming ~ 20% drop out rate. This drop-out rate is consistent with our previous studies.

### Statistical methods

The primary analysis will assess within-subject change in HEI score (3-month minus baseline) using a paired samples t-test if assumptions of normality and equal variance are met. If the equal variance assumptions are violated, an unequal-variance t-test will be used. If the data are non-normal, an appropriate transformation or non-parametric alternative will be applied. Exploratory post hoc analyses will assess heterogeneity of treatment effect, with the hypothesis that dietary quality will improve in the nutrition intervention group compared to contact control. Covariates (age, race/ethnicity, sex, medical history/comorbidity, medications, education, living situation, and annual household income) will be included in negative binomial regression models, analyzing changes in outcomes from baseline to 3 months per participant. Although weight loss is not an intended outcome, we will measure how the intervention may change body composition (DEXA). The same modeling strategies (negative-binomial regression, multinomial, or linear mixed effects models) will be applied to each outcome using piecewise approach, with a change point occurring at 6 months to determine whether treatment effects differ or persist after this time. Finally, we will test whether continued participation in the virtual nutrition intervention after intervention completion moderates the persistent effect of the intervention on study endpoints. Missing data will be handled using an intention-to-treat approach with multiple imputations, with 5–10 imputations recommended to account for uncertainty in missing values and to produce valid, unbiased estimates of treatment effects [57]. No interim analysis are planned.

### Data management, monitoring, and dissemination

The study coordinator will facilitate the creation and support of a project-specific database utilizing the VA REDCap data collection platform. REDCap is a secure, web-based application for building and managing online surveys and databases which will be used as a central location for data processing and management of research information for this project. All study team members will be adequately trained to use this centralized database to enter data and manage subjects. The Internal Safety Monitoring Board (ISMB) will review adverse events, enrollment numbers, patient charts/clinical summaries, procedure reports, audit reports, SOP, and consent forms at least annually or when indicated. The ISMB is independent from the funder. Any deviations from the protocol, breaches of confidentiality, and reportable adverse events will be reported to the institutional review board (IRB) and ISMB according to local policies. Continuing review will be performed by the IRB annually. Data will be disseminated via peer-reviewed publications.

## Discussion

Existing interventions to date have focused on non-minority populations and those with higher education levels, decreasing the ability to translate these interventions to other populations [58]. Few studies have examined determinants of dietary intake among older Veterans with limited physical functioning. Further, this is a population often excluded from dietary interventions due to multiple comorbidities [59]; thus, intervention strategies to improve dietary intake are lacking. We hypothesize that providing virtual nutrition and cooking demonstrations will reduce barriers to consuming a healthy diet such as taste, low health literacy, lack of preparation/cooking skills and access to healthy food among older Veterans. Additionally, we are reducing the economic burden of purchasing healthy produce, additional cooking utensils and healthy foods additions by incorporating produce delivery and providing spices and low-cost recipes. Providing tailored recipes to accommodate functional limitations of the participants and providing recipe modifications to accommodate those who live alone may also encourage participants to sustain these behaviors following intervention completion. The virtual group classes and the focus groups will allow the participants to develop a partnership where they can share their expertise and knowledge and collaborate in the process of developing strategies to improve their own health. Qualitative data will guide dissemination, identify best practices for collecting accurate dietary data among older adults, and refine program delivery. We anticipate that this protocol will show that virtual nutrition education and cooking demonstrations is an efficacious strategy to improve diet that also has potential for scalability through implementation into existing health promotion programs. In addition to improvements in dietary quality, we anticipate that changes in diet may be accompanied by improvements in physical function, frailty, and psychosocial outcomes, given the established links between diet quality, muscle health, and mobility [16–18]. Improvements in social engagement and perceived support through group-based participation may further reinforce dietary behavior change and contribute to enhanced quality of life among participants.

This study builds upon prior work demonstrating associations between diet quality, mobility, and social isolation, but is novel in testing a virtual, interdisciplinary intervention specifically designed for older adults with mobility limitations. By integrating nutrition education, cooking skills, occupational therapy, and social engagement, this intervention addresses multiple interrelated determinants of dietary behavior that are often studied in isolation.

There are several potential barriers to implementation that warrant mention. Despite efforts to support technology access and literacy, comfort with virtual platforms may vary across participants. Health challenges, caregiving responsibility, differences in baseline cooking skills, or home environments may affect adherence. Tracking intervention adaptations using the core functions and forms framework [35] will help identify strategies to optimize adherence and scalability.

Key limitations include reliance on self-reported dietary intake, which may be subject to recall bias, and the restriction of the sample to older Veterans in a single geographic region, which may limit generalizability. Additionally, while the study includes short-term follow up (3 months post intervention), long-term sustainability of changes in dietary quality cannot be determined.

## Data Availability

No datasets were generated or analysed during the current study.
